# Relationship between Selected Serum Metallic Elements and Obesity in Children and Adolescent in the U.S.

**DOI:** 10.3390/nu9020104

**Published:** 2017-02-03

**Authors:** Yun Fan, Chunlan Zhang, Jin Bu

**Affiliations:** 1 State Key Laboratory of Reproductive Medicine, Institute of Toxicology, Nanjing Medical University, Nanjing 211166, Jiangsu, China; yunfan_njmu@163.com (Y.F.); chunlanzhang@njmu.edu.cn (C.Z.); 2 Key Laboratory of Modern Toxicology of Ministry of Education, School of Public Health, Nanjing Medical University, Nanjing 211166, Jiangsu, China; 3 Editorial Department of Journals of Nanjing Medical University, Nanjing Medical University, Nanjing 211166, Jiangsu, China

**Keywords:** cross-sectional, metallic elements, children obesity

## Abstract

The prevalence of obesity has increased at an alarming rate worldwide. Metallic elements are involved in the pathogenesis of obesity and related diseases. To date, whether environmental exposure to metallic elements has effects on obesity in children and adolescents is still unclear. The aim of the current study was to investigate the association of blood metallic elements with obesity in U.S. children and adolescents. This cross-sectional study was performed with 5404 children and adolescents (6–19 years, 2745 males and 2659 females) who participated in the US National Health and Nutrition Examination Survey 2011–2014. Blood lead, mercury, selenium, manganese, copper, and zinc, as well as biochemical parameters including triglyceride (TG), cholesterol, low-density lipoprotein (LDL), and homeostasis model assessment of insulin resistance (HOMA-IR) were assessed for all subjects. Multivariate logistic regression and linear regression were applied to assess associations of metallic elements and overweight, obesity status, and serum metabolites as distinct outcomes adjusted for age, gender, ethnicity, and the poverty income ratio. When stratified by age and sex, significant associations were found between the highest quartile of copper concentrations in blood with obesity status (OR = 9.27, 95% CI: 5.43, 15.82, *p*_for trend_ < 0.001) and cholesterol (OR = 3.08, 95% CI: 1.43, 6.63, *p*_for trend_ < 0.001). The highest concentrations of manganese in the blood was associated with obesity in those aged 6–19 years (OR = 2.29, 95% CI: 1.74, 3.02, *p*_for trend_ < 0.001). Moreover, blood mercury and selenium showed positive relationships with cholesterol. Further, a negative association existed between blood zinc and obesity. The National Health and Nutrition Examination Survey data provide epidemiological evidence that blood metallic elements are positively associated with obesity in children and adolescents. However, the underlying mechanisms still need further exploration.

## 1. Introduction

Overweight and obesity is defined as excessive fat accumulation that presents risks to human health, which has become one of the major risk factors for a range of chronic diseases, including diabetes [1], cardiovascular disease [2], and cancer [3]. Sixty-five percent of the world’s population live in a country where overweight and obesity kills more people than underweight. Globally, 44% of diabetes, 23% of ischemic heart disease, and 7%–14% of certain cancers are attributable to overweight and obesity, and the onset of adult diseases caused by obesity has an increasing trend towards earlier age. Childhood obesity has been one of the most serious public health challenges in the 21st century [4], affecting the development of the quality of the global population. The prevalence of obesity has increased at an alarming rate. In 2013, the number of overweight children younger than 5 years of age was estimated to be more than 42 million globally. There are various factors affecting overweight and obesity, including genetic factors, environmental factors, lifestyle preferences, and cultural factors [5,6]. 

Metallic elements generally exist in the environment and in food. Although the metallic elements in the human body are usually in small quantities, some of them play important biological roles in enzymes, hormones, vitamins, and normal metabolism [7,8]. A lack of essential metallic elements causes abnormal physiological functions and organizational structure, and thus eventually leads to a variety of related diseases and disorders. For example, low manganese levels may destabilize DNA, decrease protein synthesis, and lead to mitochondrial dysfunction [9,10]. A recent study suggests that deficiency in some metallic elements may be associated with the incidence of obesity and an increase of fat deposition [11]. Moreover, an excess of any of the metallic elements is harmful for the body. However, the literature on metallic elements status in child obesity is insufficient.

Therefore, we conducted this study to investigate the associations between metallic elements and obesity in children and adolescents. We used the cross-sectional data from the National Health and Nutrition Examination Surveys (NHANES) to investigate the relationships between serum metallic elements (Cu, Zn, Mn, Pb, Hg, and Se) and body mass index (BMI), triglyceride, low density lipoprotein cholesterol (LDL-C), cholesterol, insulin, and the homeostasis model assessment of insulin resistance index (HOMA-IR) in 5404 children and adolescent subjects 6–19 years of age, and further to explore the potential association between blood metallic element status and obesity in people aged 6 to 19 years old. 

## 2. Materials and Methods

### 2.1. Study Population

NHANES is a nationally representative cross-sectional survey conducted by the US National Center for Health Statistics (Centers for Disease Control and Prevention, Atlanta, GA, USA). The data selected in the present study were obtained from two cycles of NHANES (2011–2012, 2013–2014). We used information from the questionnaire, laboratory, diet, and physical examination components of the NHANES, which included blood collection. We examined the association of serum metallic elements with obesity and metabolism-related indicators in children and adolescents who participated in the NHANES [12,13,14,15]. Subjects aged 6–19 years in the NHANES 2011–2014 were selected from a random subgroup for the measurement of serum metals. The final study sample consisted of 5404 children and adolescents (6–19 years of age, 2745 males and 2659 females).

### 2.2. Measurement of Serum Metals and Serum Metabolites

Whole blood samples were processed, stored, and shipped to the National Center for Environmental Health, and Centers for Disease Control and Prevention for analysis. Specimens were stored at −70 °C until tests. During the dilution step, a small volume of whole blood was separated from the specimen after vortexing of the entire specimen to ensure a uniform distribution of components. The elaborate laboratory methodologies are available on the website [16]. In brief, tetramethylammonium hydroxide (TMAH, 0.4% *v*/*v*), Triton X-100TM (0.05%), and ammonium pyrrolidine dithiocarbamate (APDC) (0.01%) were used to solubilize blood components and metals. Liquid samples were measured by inductively coupled plasma mass spectrometry (ICP-MS). For the measurement of blood lead, cadmium, total mercury, and manganese, the internal standards rhodium, iridium, and tellurium were at a constant concentration in all blanks, calibrators, quality control, and samples. For the detection of serum zinc, copper, and selenium, isotopes were detected by ICP-MS as zinc (*m*/*z* 64), copper (*m*/*z* 65), and selenium (*m*/*z* 78), and the internal standard gallium (*m*/*z* 71). Serum samples were diluted with an equivalent volume of water and 28-fold volume diluent containing Ga for multi-internal standardization. According to a previous study, we used abnormal lipid cut points from the Adult Treatment Panel (ATP) for each outcome in adolescents. These values included triglycerides of 150 mg/dL for both males and females; total cholesterol of 200 mg/dL for both males and females; and LDL-C of 130 mg/dL for both males and females [17]. To assess insulin resistance as a categorical outcome, we used the cut point of 4.39 units of HOMA-IR. This cut point was chosen based on a previous study that examined the distribution and associations of HOMA-IR with sex, race/ethnicity, age, and weight status as measured by BMI among U.S. adolescents in the NHANES [18].

### 2.3. Evaluation of Fatness: BMI

BMI—the index of weight (kg) divided by the square of height (m^2^)—is commonly used to assess underweight, normal weight, overweight, or obese. We defined children and adolescents as overweight and obese based on sex- and gender-cut-off points [19]. 

### 2.4. Covariates

The ratio of family income to poverty threshold was designed to reflect socio-economic status, abbreviated as poverty:income ratio (PIR). In light of the U.S. Department of Agriculture calorie intake guidelines [20], individual daily energy intake was divided into “normal” and “excessive” by gender and age. According to previous reports on factors associated with child obesity, self-reported television watching and computer games were cut off as <2 or ≥2 h/day [21].

### 2.5. Statistical Analysis

Multinomial logistic regression models were used to examine the relationships of overweight and obesity status and serum metabolites as distinct outcomes. The lowest concentration (quartile 1) was used as the reference value. We performed multiple linear regressions to explore the associations between serum metallic elements and BMI, serum metabolites (continuous variables) stratified by age (6–12 years; 13–19 years), and gender (male and female). Several confounding factors, such as age, race, PIR, TV, computer, and video game use, and caloric intake, were adjusted in multinomial logistic regressions and multiple linear regressions. All statistical analyses were performed using the Statistical Analysis Systems software package version 9.2 (SAS Institute, Inc., Cary, NC, USA). A *p*-value < 0.05 was considered as statistically significant.

## 3. Results

The subjects consisted of 5404 children and adolescents aged 6 to 19 years old, and were divided into two age groups (6–12 years and 13–19 years) and two gender groups (male and female). 

Table 1 presents the baseline characteristics and mean concentrations of serum metabolite levels among the participants. We evaluated the distribution of serum levels of metallic elements in male children. In total, we included 4021 subjects for the detection of blood lead, cadmium, mercury, selenium, and manganese, and selected 1500 individuals participating in serum copper and zinc detection (Table S1). 

In addition, we analyzed the correlation among metallic elements (Table S2), and found that no clinically significant autocorrelations (Spearman: −0.101 < *r* < 0.125) occurred among the measured concentrations of lead, cadmium, mercury, selenium, manganese, and copper.

A multivariate logistic regression analysis was performed to evaluate the association between metallic elements (lead, mercury, selenium, manganese, copper, and zinc) and BMI, triglycerides, cholesterol, LDL-C, and HOMA-IR. Table 2 shows significant correlations between mercury, selenium, manganese, copper, and zinc and obesity indicators (*p* < 0.001). Notably, the highest quartile of copper concentrations in blood was associated with obesity status (OR = 9.27, 95% CI: 5.43, 15.82, *p*_for trend_ < 0.001) and cholesterol (OR = 3.08, 95% CI: 1.43, 6.63, *p*_for trend_ < 0.001). Similarly, a positive relationship of obesity with the high concentrations of manganese was observed in the 6–19 years of age group (OR = 2.29, 95% CI: 1.74, 3.02, *p*_for trend_ < 0.001). We divided the subjects into the 6–12 and 13–19 years of age groups, and each age group was divided into the male and female subgroups, and linear regression analysis was performed for associations between BMI and serum metabolites and metals. Table 3 shows that in the 6–12 years-of-age group, significant positive correlation coefficients were found in the relationships of mercury–cholesterol (*p*_for trend_ = 0.001, Female: *β* = 0.017, 95% CI: 0.002, 0.032), selenium–cholesterol (*p*_for trend_ = 0.014, Male: *β* = 0.152, 95% CI: 0.056, 0.249; Female: *β* = 0.135, 95% CI: 0.034, 0.236), manganese–BMI (*p*_for trend_ < 0.001, Male: *β* = 0.084, 95% CI: 0.046, 0.121; Female: *β* = 0.080, 95% CI: 0.039, 0.122), copper-BMI (*p*_for trend_ < 0.001, Male: *β* = 0.318, 95% CI: 0.204, 0.433; Female: *β* = 0.381, 95% CI: 0.266, 0.495), and copper–cholesterol (*p*_for trend_ < 0.001, Male: *β* = 0.131, 95% CI: 0.021, 0.241). Significant negative correlation coefficients were found in the relationship of zinc–BMI (*p*_for trend_ = 0.014, Female: *β* = −0.159, 95% CI: −0.290, −0.027). In the 13–19 years-of-age group, significant positive correlation coefficients were found in the relationships of mercury–cholesterol (*p*_for trend_ = 0.001, Male: *β* = 0.022, 95% CI: 0.003, 0.041; Female: *β* = 0.027, 95% CI: 0.008, 0.045), selenium–cholesterol (*p*_for trend_ = 0.014, Female: *β* = 0.191, 95% CI: 0.072, 0.310), copper–BMI (*p*_for trend_ < 0.001, Male: *β* = 0.471, 95% CI: 0.343, 0.600; Female: *β* = 0.326, 95% CI: 0.209, 0.442), and copper–cholesterol (*p*_for trend_ < 0.001, Female: *β* = 0.120, 95% CI: 0.026, 0.214). Significant negative correlation coefficients were found in the zinc–BMI relationships (*p*_for trend_ = 0.014, Male: *β* = −0.161, 95% CI: −0.315, −0.007; Female: *β* = −0.184, 95% CI: −0.343, −0.025).

## 4. Discussion

This is one of the first cross-sectional analyses to evaluate the association between obesity and metallic elements among U.S. children aged 6–19 years. We divided the samples into two age groups (6–12 and 13–19 years of age), and each age group was divided into two sex subgroups (male and female). We found that the increase of concentrations of blood copper and manganese was associated with obesity. In addition, mercury and selenium in females had positive correlations with cholesterol in the 13–19-years-old group. A positive association was observed between zinc and an increase of cholesterol in males in both age groups. 

The consequences of changes in metallic elements (mainly deficiency) in children were much more important for the health of the public than is generally realized [22]. The deficiency or excess of minerals has been linked to metabolic disorders such as obesity [23,24,25]. 

Zinc is an essential metallic element in many biochemical reactions, including protein production [26]; for example, it plays a major role in insulin action and insulin receptor tyrosine kinase (IRTK) activity [27,28,29], and is involved in the stabilization of insulin hexamers and the storage of hormone in the pancreas [30]. Additionally, it is also an efficient antioxidant [31]. In research with adult subjects, low concentrations of zinc were proved to be associated with higher abdominal fat [32]. Zn-a2-glycoprotein (ZAG) is an adipokine which stimulates energy expenditure in skeletal muscle and brown adipose tissue, resulting in reductions in body weight, glycaemia, triglycerides, and non-esterified fatty acids(NEFAs)found in ob/ob mice. Its level is lower in subcutaneous and visceral adipose tissue and livers of obese humans, and interestingly does not appear to be related to insulin resistance [33]. In obese individuals, low ZAG gene expression may play an important role in the development of obesity, which is associated with low serum adiponectin and high plasma leptin levels. The influence zinc has on adipocytes through the expression of leptin by promoting free fatty acid release and glucose uptake may be controlled through the expression of a number of zinc transporters in the adipocyte, which may be altered in obesity [34,35]. Our study showed a positive association between zinc and increasing cholesterol in males in both two age groups. Meanwhile, high-density lipoprotein cholesterol (HDL-C) had an inverse relationship with serum zinc concentration in both men and women. Although a positive correlation was reported between serum zinc level and HDL-C [36], a negative association was determined in a longitudinal study [37]. In addition, zinc supplementation reduced the level of HDL-C [38,39]. While the above results were inconsistent, DiMartino et al. [40] reported that the BMI of the study population was significantly higher than that of the control group, and serum zinc content was significantly lower than that of the control group. Marreiro et al. [41] showed that zinc concentrations in plasma and erythrocytes were significantly lower in the obese group, and they also found that zinc supplementation was able to improve insulin resistance in women [42]. In contrast, Weisstaub et al. [43] reported that there were no relationships between serum zinc levels and body weight in preschool children. In our study, we found that zinc status had a negative correlation with obesity, which is in accordance with some of the above studies. Further studies with large sample size are needed to explore the causality of these relationships.

Recent interest in determining copper concentration has been aroused by metabolic disorders such as obesity, and the interest in the role that deficiency/excess of metallic elements may play in these disease processes [23]. Copper—the third most abundant essential transition metal in the human body—is a component of antioxidant enzymes that act to protect the organism against free radicals. An imbalance in the metabolism of copper might trigger hypercholesterolemia and disorders in oxidative stress [44]. It has been verified that increased Cu-Zn superoxide dismutase (SOD) as well as total circulating copper [45] was detected in obese children [24]. According to the study by Lima et al. [46], the plasma copper concentration in the overweight and obese male groups was significantly higher than that in the control group. Additionally, studies have reported significantly higher serum copper levels in obese children and adolescents compared to the control groups [45,47,48]. There is a positive correlation between serum leptin and serum copper levels [49]. In our study, the rising concentration of blood copper is associated with obesity, and zinc has a negative correlation in the two age groups. There is an antagonistic effect between zinc and copper (the best known interacting metallic elements [45]), which is consistent with the results of our study and may also apply in obese children. Various factors have been proposed to possibly affect plasma levels of copper, including age. Plasma copper levels are higher in the elderly than among the younger population, which might explain the reason for a susceptible tendency in the 13–19 year-old children on a positive correlation between copper and triglycerides, cholesterol, and HOMA-IR in our study.

Few studies have investigated manganese levels in serum or different body materials in obese individuals. Our study found that the rising concentration of blood manganese is associated with obesity. At present, we cannot explain the finding with our present state of knowledge, though we know that manganese is one of the antioxidant nutrients which makes up metalloenzymes and plays a role in the metabolism of carbohydrates, protein, and lipids [50]. This may be clarified with wider ranging studies.

Mercury is a common environmental pollutant and toxicant which is not necessary in the biological processes of our body. People are continuously exposed to mercury, which is mainly produced from fossil fuel combustion, mining, and incineration plants, as well as from natural sources such as the Earth’s crust and volcanoes. Without occupational exposure to mercury, the primary source of mercury is the diet—especially fish and shellfish in the general population. In a previous study, the level of blood mercury was significantly influenced by age, sex, smoking and alcoholic drinking habits, residence area, and fish intake before blood sampling [51]. In the work of Chang et al. [52] and You et al. [53], BMI and waist circumference increased in relation to the blood mercury level. In a previous study by Meltzer et al. [54], a positive correlation of LDL-C with dietary mercury and a negative relation of HDL-C with dietary mercury were reported, while no significant association of blood mercury was observed with LDL-C in the present study. In our study, regardless of age and gender, mercury was positively correlated with an increase of total cholesterol. Batariova et al. [55], Passos et al. [56], Reis et al. [57], Caldwell et al. [58], and You et al. [53] have reported no gender difference in blood mercury level, which agrees with our findings. Some previous studies reported that mercury exposure might increase the risk of metabolic syndrome (MS) [52,59], which is a syndrome encompassing obesity, hypertension, low HDL-C, high triglyceride, and increased fasting glucose, and a three-fold increased risk of cardiovascular diseases [60]. Although a possible relationship between the dysregulation of lipid and glucose metabolism and mercury has been suggested, the mechanism of obesity by mercury exposure is not yet clear [53,60]. Oxidative stress processes may play an important role in MS (including obesity) by increasing lipid peroxidation and decreasing the antioxidant defense system [61,62,63,64].

To our knowledge, this is the one of the few investigations of an association between childhood obesity and blood metallic elements in the United States, which may add knowledge to the worldwide literature on this topic. However, there are some limitations to our study. Firstly, due to the cross-sectional design, our study prevented us from unequivocally determining the causations of the observed associations. Secondly, although we adjusted for potential confounders, some confounders, such as smoke and genetic factors were not recorded for the adjustment in the analysis.

## 5. Conclusions

In summary, our study demonstrated significant relationships between metallic elements (including blood Zn, Cu, Mn, and Hg) and obesity (such as BMI, LDL-C, triglyceride, total cholesterol, and HOMA-IR) among a population aged 6–19 years. However, the relationship between child obesity and metallic elements could be different in obese populations with an elevated BMI or changes in other obesity biochemical parameters. Although it is not possible to determine what mechanisms underlie the association between selected metallic elements and child obesity in one study, our results may provide a better understanding of childhood obesity from an epidemiological standpoint. As we all know, the age range of 6–19 years is an important period for the development of childhood obesity or comorbidities. Future studies are needed to understand the causes and consequences of metallic element status on obesity and comorbidities.

## Figures and Tables

**Table 1:** Characteristics of children and adolescents by age and gender in National Health and Nutrition Examination Surveys (NHANES) 2011–2014.

Characteristic	6–12 Years Old		13–19 Years Old	
	Male	Female	Male	Female
*n*	1580	1474	1165	1185
Age	8.8 (0.1)	8.9 (0.1)	15.9 (0.1)	15.9 (0.1)
Body Mass Index (BMI)	18.9 (0.1)	19.4 (0.1)	24.3 (0.2)	24.6 (0.2)
Waist Circumference	65.8 (0.3)	67.2 (0.4)	83.2 (0.5)	82.4 (0.4)
Serum metabolites				
Triglyceride (mmol/L)	0.78 (0.06)	0.93 (0.07)	0.92 (0.03)	0.87 (0.02)
LDL-cholesterol (mmol/L)	2.25 (0.07)	2.24 (0.08)	2.18 (0.03)	2.34 (0.03)
Fasting Glucose (mmol/L)	5.37 (0.09)	5.15 (0.05)	5.32 (0.03)	5.09 (0.03)
Total Cholesterol( mmol/L)	4.11 (0.02)	4.14 (0.02)	3.94 (0.02)	4.17 (0.02)
Insulin (pmol/L)	81.4 (6.3)	108.6 (9.5)	84.6 (3.7)	90.0 (3.1)
HOMA-IR	2.80 (0.2)	2.93 (0.1)	3.60 (0.4)	2.95 (0.1)
Race				
Mexican American	307 (19.4)	315 (21.4)	238 (20.4)	247 (20.8)
Other Hispanic	184 (11.6)	151 (10.2)	110 (9.4)	133 (11.2)
Non-Hispanic White	418 (26.5)	348 (23.6)	286 (24.5)	269 (22.7)
Non-Hispanic Black	432 (27.3)	417 (28.3)	321 (27.6)	320 (27.0)
Non-Hispanic Asian	148 (9.4)	140 (9.5)	149 (12.8)	158 (13.3)
Other Race (including Multi-Racial)	91 (5.8)	103 (7.0)	61 (5.2)	58 (4.9)
Ratio of family income to poverty				
<1	534 (36.4)	515 (37.3)	364 (34.4)	399 (37.0)
≥1	935 (63.6)	866 (62.7)	694 (65.6)	678 (63.0)
TV, computer, and video games use				
≤2	654 (42.0)	768 (52.6)	356 (31.6)	401 (35.2)
>2	903 (58.0)	692 (47.4)	772 (68.4)	738 (64.8)
Caloric intake				
Normal intake	384 (32.8)	460 (40.4)	606 (66.2)	628 (67.8)
Excessive intake	788 (67.2)	680 (59.6)	310 (33.8)	298 (32.2)
Obese status				
Overweight	513 (34.1)	560 (39.7)	488 (43.1)	446 (40.0)
Obesity	230 (15.3)	257 (18.2)	202 (17.8)	220 (19.7)

N (%) and mean (standard error). Total number of subjects from some variables (e.g., Caloric intake) do not total to 5404 due to missing data. HOMA-IR: homeostasis model assessment of insulin resistance index; LDL: low-density lipoprotein.

**Table 2:** Adjusted odds ratios (ORs) (95% CIs) for obesity levels and selected serum metabolites by exposure quartile for serum metals.

Chemicals		Overweight	Obesity	Triglyceride	Cholesterol	LDL-cholesterol	HOMA-IR
Lead	Q1	Reference	Reference	Reference	Reference	Reference	Reference
	Q2	0.99 (0.82, 1.20)	0.89 (0.71, 1.13)	0.73 (0.34, 1.55)	0.97 (0.67, 1.39)	0.87 (0.36, 2.12)	0.34 (0.17, 0.71)
	Q3	**0.77 (0.63, 0.94)**	**0.68 (0.53, 0.88)**	0.47 (0.19, 1.17)	1.07 (0.74, 1.55)	0.91 (0.33, 2.46)	0.71 (0.36, 1.39)
	Q4	**0.78 (0.64, 0.96)**	**0.62 (0.47, 0.80)**	0.82 (0.34, 1.94)	1.09 (0.73, 1.61)	1.46 (0.55, 3.90)	0.56 (0.26, 1.23)
	*p* for trend	0.018	<0.001	0.209	0.703	0.434	0.300
Mercury	Q1	Reference	Reference	Reference	Reference	Reference	Reference
	Q2	1.17 (0.97, 1.42)	1.20 (0.95, 1.53)	0.81 (0.34, 1.97)	0.99 (0.66, 1.50)	0.80 (0.32, 2.02)	0.59 (0.29, 1.19)
	Q3	1.07 (0.89, 1.30)	0.98 (0.77, 1.25)	1.61 (0.72, 3.63)	**1.55 (1.07, 2.26)**	0.66 (0.25, 1.73)	0.77 (0.39, 1.51)
	Q4	0.95 (0.77, 1.16)	1.09 (0.84, 1.41)	1.02 (0.43, 2.44)	**1.73 (1.18, 2.52)**	0.74 (0.29, 1.90)	0.47 (0.23, 0.98)
	*p* for trend	0.627	0.593	0.830	0.001	0.893	0.134
Selenium	Q1	Reference	Reference	Reference	Reference	Reference	Reference
	Q2	1.04 (0.86, 1.27)	0.94 (0.74, 1.21)	2.46 (0.82, 7.42)	1.18 (0.80, 1.76)	2.33 (0.45, 11.95)	1.77 (0.77, 4.10)
	Q3	1.11 (0.91, 1.34)	1.02 (0.80, 1.31)	1.25 (0.42, 3.69)	1.44 (0.98, 2.11)	4.34 (0.96, 19.63)	1.38 (0.61, 3.10)
	Q4	**1.23 (1.00, 1.51)**	1.14 (0.88, 1.48)	1.64 (0.57, 4.67)	**1.57 (1.06, 2.32)**	4.03 (0.90, 18.17)	1.09 (0.49, 2.47)
	*p* for trend	0.009	0.132	0.873	0.014	0.026	0.563
Manganese	Q1	Reference	Reference	Reference	Reference	Reference	Reference
	Q2	1.19 (0.97, 1.45)	**1.40 (1.07, 1.83)**	0.75 (0.29, 1.93)	0.71 (0.48, 1.06)	0.38 (0.12, 1.23)	1.15 (0.55, 2.39)
	Q3	**1.41 (1.15, 1.73)**	**1.59 (1.21, 2.08)**	1.78 (0.77, 4.16)	1.02 (0.70, 1.48)	1.27 (0.51, 3.16)	0.91 (0.43, 1.92)
	Q4	**1.69 (1.37, 2.10)**	**2.29 (1.74, 3.02)**	1.59 (0.65, 3.86)	1.10 (0.74, 1.63)	1.35 (0.54, 3.36)	0.98 (0.46, 2.09)
	*p* for trend	<0.001	<0.001	0.383	0.325	0.359	0.879
Copper	Q1	Reference	Reference	Reference	Reference	Reference	Reference
	Q2	**1.58 (1.11, 2.25)**	**2.15 (1.23, 3.77)**	1.44 (0.43, 4.83)	1.31 (0.59, 2.92)	1.64 (0.32, 8.32)	1.07 (0.33, 3.44)
	Q3	**3.14 (2.21, 4.47)**	**5.76 (3.41, 9.75)**	1.75 (0.47, 6.51)	**2.91 (1.40, 6.05)**	2.88 (0.54, 15.50)	1.69 (0.55, 5.22)
	Q4	**4.70 (3.24, 6.82)**	**9.27 (5.43, 15.82)**	0.77 (0.15, 3.89)	**3.08 (1.43, 6.63)**	3.75 (0.61, 23.03)	2.43 (0.70, 8.43)
	*p* for trend	<0.001	<0.001	0.684	<0.001	0.023	0.084
Zinc	Q1	Reference	Reference	Reference	Reference	Reference	Reference
	Q2	0.83 (0.61, 1.13)	0.92 (0.63, 1.35)	3.32 (0.30, 36.80)	1.13 (0.60, 2.10)	3.03 (0.32, 29.01)	1.15 (0.29, 4.56)
	Q3	**0.58 (0.42, 0.79)**	**0.62 (0.41, 0.92)**	4.98 (0.48, 51.47)	1.17 (0.62, 2.22)	1.50 (0.15, 14.86)	0.78 (0.18, 3.29)
	Q4	**0.65 (0.47, 0.89)**	0.73 (0.49, 1.09)	5.44 (0.56, 53.09)	1.25 (0.67, 2.36)	1.41 (0.15, 13.54)	0.99 (0.25, 3.92)
	*p* for trend	0.001	0.014	0.144	0.106	0.760	0.923

ORs were adjusted for age (years); gender (boys and girls); race (Mexican American, Other Hispanic, Non-Hispanic White, Non-Hispanic Black, Non-Hispanic Asian, and other race); poverty income ratio (<1, ≥1); TV, computer, and video games use in hours (≤2, >2); BMI (tertiles). Triglycerides pass through 150 mg/dL for males and 150 mg/dL for females. Total cholesterol passes through 200 mg/dL for males and 200 mg/dL for females. LDL cholesterol passes through 130 mg/dL for males and 130 mg/dL for females. HOMA-IR passes through 4.39 units for males and 4.39 units for females.

**Table 3:** Adjusted regression coefficient (β) and 95% Confidence Intervals (95% CI) in BMI and serum metabolites and serum metals stratified by age and gender.

			BMI	Triglyceride	LDL-Cholesterol	Total Cholesterol	Insulin	HOMA-IR
6–12	Male	Lead	**−0.023 (−0.043, −0.002)**	−0.092 (−0.388, 0.205)	**0.128 (0.013, 0.244)**	0.009 (−0.009, 0.028)	0.092 (−0.139, 0.322)	0.120 (−0.132, 0.372)
		Mercury, total	0.010 (−0.006, 0.027)	−0.119 (−0.362, 0.125)	−0.021 (−0.118, 0.075)	0.014 (−0.001, 0.029)	0.130 (−0.058, 0.319)	0.122 (−0.086, 0.33)
		Selenium	−0.077 (−0.187, 0.033)	0.258 (−1.159, 1.675)	0.194 (−0.382, 0.771)	**0.152 (0.056, 0.249)**	0.316 (−0.784, 1.416)	0.207 (−1.021, 1.436)
		Manganese	**0.084 (0.046, 0.121)**	0.325 (−0.228, 0.877)	0.122 (−0.104, 0.348)	−0.006 (−0.040, 0.028)	0.120 (−0.315, 0.555)	0.204 (−0.278, 0.686)
		Copper	**0.318 (0.204, 0.433)**	−1.120 (−2.560, 0.320)	0.196 (−0.227, 0.620)	**0.131 (0.021, 0.241)**	0.077 (−1.125, 1.279)	−0.088 (−1.446, 1.269)
		Zinc	−0.051 (−0.166, 0.064)	−0.162 (−1.419, 1.094)	0.205 (−0.141, 0.551)	**0.159 (0.057, 0.261)**	−0.869 (−1.785, 0.047)	**−1.082 (−2.097, −0.066)**
	Female	Lead	−0.003 (−0.030, 0.024)	−0.045 (−0.345, 0.255)	0.085 (−0.180, 0.350)	0.018 (−0.004, 0.041)	0.142 (−0.147, 0.431)	0.147 (−0.148, 0.441)
		Mercury, total	−0.005 (−0.024, 0.013)	0.158 (−0.047, 0.363)	−0.063 (−0.198, 0.072)	**0.017 (0.002, 0.032)**	−0.009 (−0.211, 0.192)	−0.039 (−0.244, 0.167)
		Selenium	0.054 (−0.070, 0.178)	0.763 (−0.459, 1.985)	−0.198 (−1.283, 0.887)	**0.135 (0.034, 0.236)**	0.190 (−1.004, 1.383)	0.200 (−1.017, 1.416)
		Manganese	**0.080 (0.039, 0.122)**	0.270 (−0.083, 0.623)	−0.050 (−0.366, 0.266)	−0.004 (−0.038, 0.030)	−0.129 (−0.475, 0.217)	−0.120 (−0.473, 0.233)
		Copper	**0.381 (0.266, 0.495)**	**−1.159 (−2.278, −0.41)**	−0.488 (−1.488, 0.512)	0.065 (−0.036, 0.167)	0.078 (−0.908, 1.064)	0.122 (−0.884, 1.128)
		Zinc	**−0.159 (−0.290, −0.027)**	−0.105 (−1.809, 1.599)	0.397 (−0.931, 1.725)	**0.138 (0.033, 0.243)**	−0.264 (−1.533, 1.005)	−0.177 (−1.48, 1.126)
13–19	Male	Lead	**−0.032 (−0.064, 0.0)**	−0.010 (−0.117, 0.097)	0.032 (−0.028, 0.093)	0.004 (−0.023, 0.031)	−0.070 (−0.190, 0.050)	−0.068 (−0.196, 0.060)
		Mercury, total	−0.003 (−0.025, 0.020)	−0.015 (−0.090, 0.060)	0.017 (−0.027, 0.060)	**0.022 (0.003, 0.041)**	−0.008 (−0.094, 0.078)	−0.011 (−0.102, 0.08)
		Selenium	**0.171 (0.016, 0.326)**	0.292 (−0.203, 0.787)	0.239 (−0.046, 0.525)	0.101 (−0.031, 0.232)	0.102 (−0.451, 0.655)	0.082 (−0.507, 0.671)
		Manganese	0.015 (−0.041, 0.072)	0.170 (−0.020, 0.360)	0.067 (−0.045, 0.179)	**0.050 (0.002, 0.098)**	0.140 (−0.073, 0.352)	0.166 (−0.061, 0.393)
		Copper	**0.471 (0.343, 0.600)**	0.158 (−0.036, 0.352)	0.166 (−0.113, 0.445)	0.083 (−0.039, 0.205)	0.523 (−0.051, 1.098)	**0.604 (0.000, 1.208)**
		Zinc	**−0.161 (−0.315, −0.007)**	**0.572 (0.092, 1.052)**	**0.436 (0.122, 0.750)**	**0.150 (0.025, 0.276)**	−0.062 (−0.725, 0.601)	0.036 (−0.663, 0.735)
	Female	Lead	**−0.077 (−0.122, −0.031)**	0.046 (−0.104, 0.196)	0.004 (−0.089, 0.097)	0.020 (−0.016, 0.056)	−0.039 (−0.195, 0.117)	−0.032 (−0.192, 0.128)
		Mercury, total	−0.022 (−0.046, 0.002)	0.052 (−0.018, 0.121)	0.020 (−0.021, 0.061)	**0.027 (0.008, 0.045)**	0.037 (−0.036, 0.109)	0.042 (−0.033, 0.116)
		Selenium	0.026 (−0.130, 0.183)	**0.447 (0.018, 0.877)**	**0.331 (0.069, 0.593)**	**0.191 (0.072, 0.310)**	0.109 (−0.348, 0.567)	0.079 (−0.391, 0.549)
		Manganese	−0.020 (−0.075, 0.034)	0.136 (−0.021, 0.293)	0.037 (−0.060, 0.134)	0.039 (−0.003, 0.080)	0.059 (−0.109, 0.226)	0.076 (−0.096, 0.248)
		Copper	**0.326 (0.209, 0.442)**	0.296 (−0.161, 0.752)	0.175 (−0.093, 0.443)	**0.120 (0.026, 0.214)**	0.249 (−0.193, 0.691)	0.263 (−0.194, 0.721)
		Zinc	**−0.184 (−0.343, −0.025)**	**0.663 (0.115, 1.211)**	0.054 (−0.274, 0.382)	0.110 (−0.009, 0.229)	0.225 (−0.315, 0.764)	0.210 (−0.349, 0.768)

BMI, serum metabolites, and metals were Ln-transformed in models. Models were adjusted for age, race, poverty income ratio, TV, computer and video games use hour (bivariate), and BMI.

## Supplementary Materials

The following are available online at http://www.mdpi.com/2072-6643/9/2/104/s1. Table S1. Distribution of serum metal concentrations in children and adolescents in National Health and Nutrition Examination Surveys (NHANES) 2011–2014, Table S2. Spearman correlation between different serum metals.

## Abbreviations

WHO	World Health Organization
NHANES	National Health and Nutrition Examination Surveys
BMI	body mass index
LDL	low-density lipoprotein
LDL-C	low-density lipoprotein cholesterol
HOMA-IR	insulin resistance index
TMAH	tetramethylammonium hydroxide
APDC	ammonium pyrrolidine dithiocarbamate
ATP	Adult Treatment Panel
ICP-MS	inductively coupled plasma mass spectrometery
PIR	poverty:income ratio
IRTK	insulin receptor tyrosine kinase
ZAG	zinc-alpha2-glycoprotein
NEFAs	non-esterified fatty acids
SOD	superoxide dismutase
TC	total cholesterol
HDL-C	high-density lipoprotein cholesterol
MS	metabolic syndrome
TG	triglyceride
Zn	zinc
Cu	copper
Mn	manganese
Pb	lead
Hg	mercury
Se	selenium
