# Supplementary material for: Relationship between Selected Serum Metallic Elements and Obesity in Children and Adolescent in the U.S
Source: Nutrients. 2017 Feb 3;9(2):104. doi: 10.3390/nu9020104 (PMC5331535; doi:10.3390/nu9020104)
Supplement: Supplementary file 1 [file nutrients-09-00104-s001.docx]

Supplementary Materials: Relationship between Selected Serum Metallic Elements and Obesity in Children and Adolescent in the U.S.

Yun Fan, Chunlan Zhang and Jin Bu

**Table S1.** Distribution of serum metal concentrations in children and adolescents in National Health and Nutrition Examination Surveys (NHANES) 2011–2014.

| **Analyte** | **Number of Detection** | **Percent > LOD** | **Concentration (Mean (Range))** |
| --- | --- | --- | --- |
| Lead (μg/dL) | 4021 | 97.0% | 0.74 (0, 15.37) |
| Cadmium (μg/L) | 4021 | 50.8% | 0.10 (0, 3.54) |
| Mercury, total (μg/L) | 4021 | 94.9% | 0.65 (0, 13.71) |
| Selenium (μg/L) | 4021 | 100% | 183.47 (109.26, 327.08) |
| Manganese (μg/L) | 4021 | 100% | 10.69 (3.21, 58.86) |
| Copper (μg/dL) | 1500 | 100% | 115.71 (32.10, 297.50) |
| Zinc (μg/dL) | 1500 | 100% | 82.65 (38.90, 198.60) |

LOD: limit of detection.

**Table S2.** Spearman correlation between different serum metals.

|  | **Lead** | **Cadmium** | **Mercury, Total** | **Selenium** | **Manganese** | **Copper** | **Zinc** |
| --- | --- | --- | --- | --- | --- | --- | --- |
| Lead | 1 | 0.055 ** | 0.060 ** | −0.070 ** | −0.040 ** | 0.009 | 0.058 ** |
| Cadmium |  | 1 | 0.099 ** | 0.064 ** | 0.065 ** | −0.059 * | −0.013 |
| Mercury, total |  |  | 1 | 0.050 ** | 0.125 ** | −0.048 | 0.038 |
| Selenium |  |  |  | 1 | 0.031 | −0.101 | 0.113 ** |
| Manganese |  |  |  |  | 1 | 0.035 | −0.018 |
| Copper |  |  |  |  |  | 1 | 0.035 |
| Zinc |  |  |  |  |  |  | 1 |

*, *p* < 0.05; **, *p* < 0.01.
